# Teaching physiotherapy skills in culturally-diverse classes

**DOI:** 10.1186/1472-6920-11-34

**Published:** 2011-06-16

**Authors:** Andrea Bialocerkowski, Cherie Wells, Karen Grimmer-Somers

**Affiliations:** 1 School of Biomedical and Health Sciences, University of Western Sydney, Locked Bag 1797, Penrith, NSW, 2751, Australia; 2 International Centre for Allied Health Evidence, University of South Australia, North Terrace Adelaide, 5000, Australia

## Abstract

**Background:**

Cultural competence, the ability to work in cross-cultural situations, has been acknowledged as a core skill for physiotherapists and other health professionals. Literature in this area has focused on the rationale for physiotherapists to provide culturally-competent care and the effectiveness of various educational strategies to facilitate the acquisition of knowledge about cultural competence by physiotherapists and physiotherapy students. However, there is a paucity of research on how students with different cultural needs, who are attending one university class, can be accommodated within a framework of learning core physiotherapy skills to achieve professional standards.

**Results:**

This paper reports on steps which were taken to resolve the specific needs of a culturally-diverse body of first year physiotherapy students, and the impact this had on teaching in a new physiotherapy program located in Greater Western Sydney, Australia. Physiotherapy legislative, accreditation and registration requirements were considered in addition to anti-discrimination legislation and the four ethical principles of decision making.

**Conclusions:**

Reflection on this issue and the steps taken to resolve it has resulted in the development of a generic framework which focuses on providing quality and equitable physiotherapy education opportunities to all students. This framework is generalizable to other health professions worldwide.

## Background

Culture has been defined as a "sharing of values, beliefs, practices and behavioural norms within a specific group of people giving them a common identity" (p.4) [1]. Understanding culture in the context of how people choose, access and respond to health care is an essential skill for health professionals [2]. This skill, termed "cultural competence", was first described as a "set of congruent behaviours, attitudes and policies that come together in a system, agency or among professionals to work effectively in cross-cultural situations" (p.iv) [3]. Since this time, many cultural competency models have been proposed for use in health professional training.

One of these models, the trans-cultural skill development model by Popadopoulos et al. [4], appears most relevant to health professional training, including physiotherapy. Popadopoulos et al. [4] suggests that cultural competence is the culmination of cultural awareness, cultural knowledge and cultural sensitivity. When this theory is applied to physiotherapy, it means that physiotherapists need to be aware of their own cultural identity; they need to possess cultural knowledge of common health beliefs and behaviours; they need to display culturally-sensitive behaviours (e.g. empathy, trust, acceptance, respect); and they need to use this knowledge and skills to modify their approach so that it meets the culturally-diverse needs of their clientele. Therefore, cultural competence can be considered as a core skill for client-centred care which has been linked to improved treatment compliance and patient outcomes.

The growing body of literature pertaining to cultural competence and physiotherapy focuses on the rationale for physiotherapists to provide culturally-competent care and the effectiveness of various educational strategies to facilitate the acquisition of knowledge about cultural competence by physiotherapists and physiotherapy students. Moreover, recommendations have been made to increase the cultural diversity of physiotherapists in predominantly Anglo-Saxon countries or regions, to reflect the changing diversity of communities that require health care. However, there is a paucity of research on improving the cultural competence of physiotherapy educators (i.e. university-based academic teaching staff and community-based clinical educators) or how students with different cultural needs, who are attending one class, can be accommodated within a framework of learning core physiotherapy skills to achieve professional standards.

Physiotherapy educators are responsible for teaching physiotherapy students how to be safe, efficient and effective health care providers in potentially multi-cultural environments. Consequently, educators should possess expertise in a core area of physiotherapy and be competent in dealing with issues concerning cultural diversity in health care. In addition, physiotherapy educators act as role models for students, and therefore require highly developed cultural awareness, cultural knowledge, cultural sensitivity and cultural competence. These skills are necessary to provide high quality physiotherapy education to all physiotherapy students, which meet the professional standards and ensures that each student develops essential physiotherapy competencies. Despite this, little attention in the research has been focused on the role of the physiotherapy educator when dealing with a culturally-diverse student body, and the subsequent impact of role adjustment on the physiotherapy curriculum and the mode of teaching.

This paper reports on steps which were taken to resolve the specific needs of a culturally-diverse body of first year physiotherapy students, and the impact this had on teaching in a new physiotherapy program located in Greater Western Sydney, Australia. This paper presents the processes which were used to resolve issues related to the cultural diversity of the student cohort, within the framework of providing quality and equitable physiotherapy education opportunities to all students.

### Background - Greater Western Sydney, the University of Western Sydney and the Physiotherapy program

Greater Western Sydney is a geographical area of 9,000 square kilometres located west of the central business district of Sydney, Australia. It is the largest manufacturing region in Australia and it has one of the fastest growing economies in the country. The population of Greater Western Sydney is rapidly expanding. Currently, one in 11 Australians reside in this area and over one third of the population is under 25 years. Moreover, the population has over 100 nationalities represented. Greater Western Sydney is home to the University of Western Sydney (UWS). This university was established in 1989 and currently educates in excess of 35,000 students from Australia and around the world. Its mission is to "bring knowledge to life" and link university activities to the development of the Western Sydney region, through community and business engagement, learning and research [5].

The UWS physiotherapy program had its first intake of students in March 2010. This program is located in the School of Biomedical and Health Sciences, which offers other health science courses, such as occupational therapy, podiatric medicine, traditional Chinese medicine, diversional therapy (therapeutic recreation) and sports and exercise science [5]. The vision of the physiotherapy program is to attract students who reside in Greater Western Sydney, to study, then practice physiotherapy in this region upon graduation. This vision supports the rapid expansion of the population in Greater Western Sydney, and its need for health care services.

The overarching UWS physiotherapy program framework overtly recognized that the multicultural community of Greater Western Sydney required diversity in healthcare professionals trained appropriately to deal with the region's diverse demographics. Therefore, attracting a culturally-diverse student cohort to study physiotherapy at the University of Western Sydney was a strategy that would have future community benefits. Indeed, the first cohort of physiotherapy students mirrored the cultural diversity in Greater Western Sydney. Twenty-eight of the 59 students (47% of the cohort) identified themselves as being from a non-Australian background. Sixteen different cultures were represented within this group, with most being from an Asian or Middle Eastern background (n = 14).

### Structure of the physiotherapy program

The structure of the UWS physiotherapy program was conceptualized to provide a progressive development of physiotherapy skills over the four years of study. In the first two years of the program, students will develop core interdisciplinary health competencies (e.g. communication skills) and medical science knowledge (e.g. anatomy, physiology, biomechanics, pathophysiology, pharmacology). Physiotherapy theory and skills are primarily taught in the university setting in Year 3 of the program, whereas in Year 4, students apply these skills in a variety of community settings under the supervision of a clinical educator. In the first semester of study, students also undertake one physiotherapy-specific unit, which provides an introduction to physiotherapy and the skills that are required by physiotherapists. It is in this unit that students first begin to develop physiotherapy-specific skills in the areas of communication, observation and palpation.

### The issues

Within the first week of the first cohort of students commencing study, it became apparent that student beliefs based on cultural background (i.e. cultural individualism) could impact on their learning, particularly in relation to the development of core physiotherapy skills (observation and palpation). Issues brought to the attention of physiotherapy educators included:

• Students not wishing to practice physiotherapy techniques on the opposite gender as some cultures forbid skin contact with the opposite gender; and

• Students not wishing to disrobe in mixed-gender classes as some cultures forbid exposure of the skin (other than the hands, feet and face) to the opposite gender. Disrobing is recognized as a key component to experience physiotherapy techniques and to allow students to practice techniques.

Traditionally, physiotherapy hands-on skills are first taught in a controlled, University environment, under the supervision of a qualified physiotherapist, i.e. a clinical educator. Students practice these skills on their peers. They are encouraged to practice on students who vary in body size, gender and cultural background, as well as to experience what it is like to have physiotherapy techniques performed on them. Therefore, students not wishing to practice physiotherapy techniques on students of the opposite gender and not wishing to disrobe in a mixed gender class could potentially impact on their ability to achieve competence in core physiotherapy practices. Moreover, without achieving these core competencies, students may be considered unsafe to commence clinical training in the community; an environment which is more unpredictable than that encountered in the University setting.

In considering how best to address the students' cultural concerns, the authors also reflected that clients in the Western Sydney may also express an unwillingness to expose their skin and may not wish to be treated by therapists of the opposite gender [6]. Therefore, students, qualified physiotherapists and physiotherapy educators at the University of Western Sydney needed to be adequately prepared to address such issues in a professional, ethical and culturally-sensitive manner, should they arise.

### Requirements of physiotherapy education

**Figure 1:**
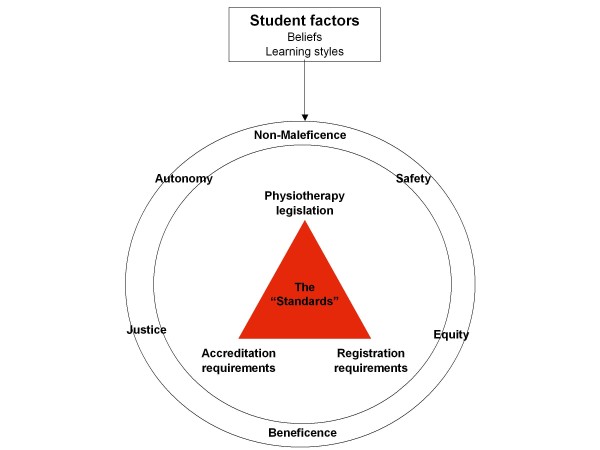
**Factors which inform physiotherapy curriculum development**.

A number of elements were important to consider when resolving these issues. First, the literature was searched to identify frameworks which could potentially be utilised to address these issues. A paucity of published literature was found on this topic. Second, consultation was undertaken with Heads of the Physiotherapy discipline in other Australian universities. This failed to identify a framework which could be adopted for this purpose. Therefore, it was considered essential to develop a framework which would facilitate the resolution of these issues as well as one which could be used to address other issues pertaining to the cultural diversity of the student cohorts, should they arise in the future. This required the consideration of the legislative, registration and accreditation requirements of the physiotherapy profession, anti-discrimination legislation/university policies, and ethical principles and how these inform curriculum development, mode of teaching and student learning outcomes. Figure 1 provides a diagrammatic representation of these demands.

### Physiotherapy legislative, accreditation and registration requirements

In many countries, the physiotherapy profession is bound by statutory regulation to assist in maintaining the scope and quality of physiotherapy practice. In Australia, the *Health Practitioner Regulation National Law Act *(2009) [7] governs health professions, such as physiotherapy. This means that there is one set of physiotherapy standards, one registration process for physiotherapists, and one accreditation process for physiotherapy training programs across the seven states and two territories in Australia. This Act is relevant to Australian physiotherapy educators as students need to demonstrate compliance with regulatory requirements during university study, as well as understanding the requirements of a qualified physiotherapist prior to graduation [8].

The Australian Standards for Physiotherapy [9] ("the Standards"), provide the physiotherapy profession with benchmarks for knowledge, skills and attributes of a safe and effective entry level physiotherapist in Australia (Table 1). These standards are the Australian equivalent to the standards of physiotherapy practice in New Zealand, the United Kingdom, the United States of America and Canada."The Standards" are embedded into the curricula of all entry level physiotherapy programs in Australia. Moreover, "the Standards" are integrated into the process of accreditation of Physiotherapy teaching programs. Graduates from accredited physiotherapy programs need to meet "the Standards" to graduate. Consequently they also satisfy the academic requirement to register to practice as a physiotherapist in Australia [9]. Therefore, it is imperative that Universities ensure that all students, irrespective of their cultural beliefs, attain "the Standards". To achieve this, Universities have policies and procedures in place, to ensure that educational standards are met and maintained irrespective of the cultural diversity of student cohorts.

**Table 1:** Australian Standards for Physiotherapy [9]

Standard		Elements	
1	Demonstrate professional behaviour appropriate to physiotherapy	1.1	Demonstrate practice that is ethical and in accordance with relevant legal and regulatory requirements
		1.2	Demonstrates strategies to maintain and extend professional competence
		1.3	Operate within individual and professional strengths and limitations
2	Communicate effectively	2.1	Communicate effectively with the client
		2.2	Adapt communication style recognising cultural safety, and cultural and linguistic diversity
		2.3	Communicate effectively with other service providers
		2.4	Prepare and deliver presentations
		2.5	Prepare and provide documentation according to legal requirements and accepted policies and procedures
3	Assess, interpret and apply information to continuously improve practice	3.1	Demonstrate a working knowledge and understanding of theoretical concepts and principles relevant to physiotherapy practice
		3.2	Apply contemporary forms of information management to relevant areas of practice
		3.3	Apply and evidence-based approach to own learning
		3.4	Acquire and apply new knowledge to continuously improve own practice
4	Assess the client	4.1	Collect client information
		4.2	Form a preliminary hypothesis
		4.3	Design and conduct an assessment
		4.4	Conduct assessment safely
5	Interpret and analyse the assessment findings	5.1	Compare findings with 'normal'
		5.2	Compare findings with what is expected for the condition, and include or exclude alternative diagnoses
		5.3	Prioritise client needs
		5.4	Re-evaluate as required, to develop a justifiable and sustainable hypothesis
		5.5	Identify areas that are outside skills and expertise and refer client appropriately
6	Develop a physiotherapy intervention plan	6.1	Develop a rationale for physiotherapy intervention
		6.2	Set realistic short and long term goals with the client
		6.3	Select appropriate intervention
		6.4	Plan for possible contingencies that may affect intervention plan
		6.5	Prioritise intervention plan in collaboration with the client
		6.6	Determine plan of evaluation that uses valid and reliable outcome measures
7	Implement safe and effective physiotherapy intervention(s)	7.1	Obtain informed consent for the intervention
		7.2	Prepare equipment and treatment area appropriate to the intervention
		7.3	Implement intervention safely and effectively
		7.4	Manage adverse events
		7.5	Provide strategies for client self management
		7.6	Implement health promotion activities
8	Evaluate the effectiveness and efficiency of physiotherapy intervention(s)	8.1	Monitor the outcomes of the intervention
		8.2	Evaluate the outcomes of the intervention
		8.3	Determine modifications to the intervention
9	Operate effectively across a range of settings	9.1	Use a model of service delivery relevant to the practice setting
		9.2	Work effectively within a team
		9.3.	Manage own work schedule to maximise safety, efficiency and effectiveness
		9.4	Operate within own role and according to responsibilities
		9.5	Participate in quality improvement processes

To practice physiotherapy in Australia, a physiotherapist must be registered. There are a number of requirements for registration including possessing a qualification from an accredited physiotherapy program in Australia or an equivalent qualification from an overseas institution [10]. Therefore, registration to practice physiotherapy in Australia requires the attainment and maintenance of a predetermined level of education.

### Antidiscrimination legislation

A review of antidiscrimination legislation is imperative when resolving issues associated with a culturally-diverse cohort. Within an academic environment, students cannot be discriminated on the grounds of race, sex, domestic status, disability or their age [11]. Therefore, meeting the requirements for entry into a course and obtaining the acquired academic standards (as dictated by legislation, accreditation and registration) within the course are the benchmarks for academic progression and ultimately graduation.

The constraints on a student when being of a specific culture (cultural individualism) may impact on their ability to attain the required academic standards because of their non-participation in academic learning activities. For example, the development of competency in the performance of physiotherapy skills often involves students practicing techniques on each other. This type of skill learning is facilitated by physiotherapy educators demonstrating techniques to the students, supervising students while they are practicing the techniques on other students and providing students with feedback on their performance. Students who act as "patients" also provide feedback on the performance of the technique to "therapist" students. These students experience what it is like to have the technique performed on them, and this information is subsequently used in the clinical setting when explaining treatments to patients.

When students raise issues related to the specific requirements of study which they cannot fulfil because of cultural grounds (such as their inability to disrobe or to touch the opposite gender), physiotherapy educators need to examine and reflect on their teaching methods to ensure that they do not exclude students from all possible learning opportunities based on their culture (i.e. inadvertent discrimination), while still achieving learning outcomes [12]. Reflecting on physiotherapy teaching methods in this manner is particularly important in a culturally-diverse cohort of students which may well have different cultural issues from one year to the next. In this situation, replicating 'usual' teaching methods year after year may not be appropriate to the student cohort and individual students' methods of learning.

**Figure 2:**
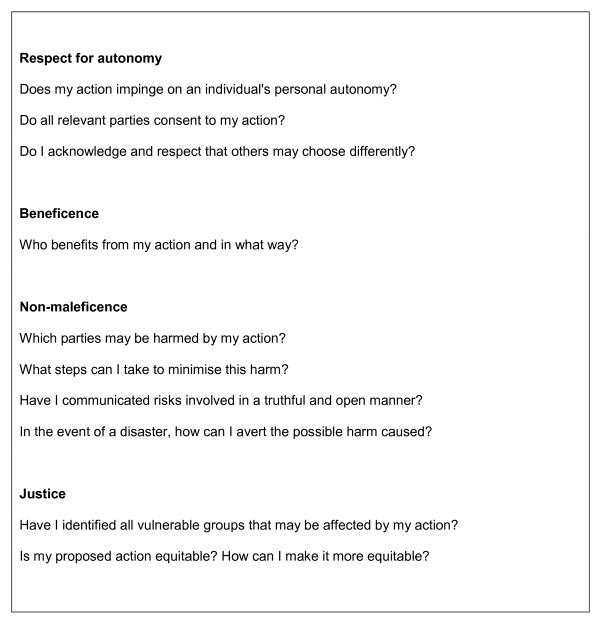
**The four principles for ethical decision-making **[13].

It is clear that "reasonable" accommodations should be made for each student on an individual basis. What is reasonable or not can be deduced by considering the four ethical principles for decision-making, which are respect for autonomy, beneficence, non-maleficence and justice [13]. The four ethical principles (Figure 2) should be applied to determine "reasonable" accommodations to resolve student issues associated with acting in a culturally-appropriate manner within the specific requirements of physiotherapy study and within the legislative, accreditation and registration requirements for a health professional. Respect for autonomy, when applied to this context, refers to the physiotherapy educators' respect of student beliefs particularly when they may be different to their own, and potentially challenging to their role as an educator. Educators need to provide students with adequate information regarding the physiotherapy legislative, accreditation and registration requirements, and ensure that students have an understanding of how these requirements translate into physiotherapy training. This is essential so that students can make informed decisions regarding their ability to meet these requirements and therefore their potential capacity to attain the physiotherapy standards and graduate as a physiotherapist.

One way of addressing autonomy is to request students to suggest possible solutions to their cultural issues affecting physiotherapy training and practice. This process engages students by placing their concerns in context of appropriate legislative, accreditation and registration frameworks, and focuses discussion away from specific physiotherapy educators or the University. This collaborative approach may depersonalize cultural issues enabling differences to be identified, considered, included and valued. Students may also be empowered to consider their personal autonomy in the context of social responsibility to their fellow students, future clients and the physiotherapy profession.

Student safety is of primary importance when designing curriculum, or when modifying aspects of curriculum to address issues of cultural individualism. When considering possible solutions to individual student issues, benefit (beneficence) needs to be weighed against risk or harm (non-maleficence), as potential solutions may affect the whole student cohort, not just individual students. It is, therefore, paramount to consider how possible solutions impact on the physical and emotional safety of the individual within the context of the student cohort. This will ensure that potential solutions are equitable (justice) across the whole cohort of students. These aspects of ethical decision-making are explicitly linked to "reasonable accommodations" in the anti-discrimination legislation. As such, students need to be made aware of how the principles of beneficence, non-maleficence and justice were applied to address their situation; the potential solutions which were considered; and the justification for adopting or not adopting potential solutions.

## Discussion

**Figure 3:**
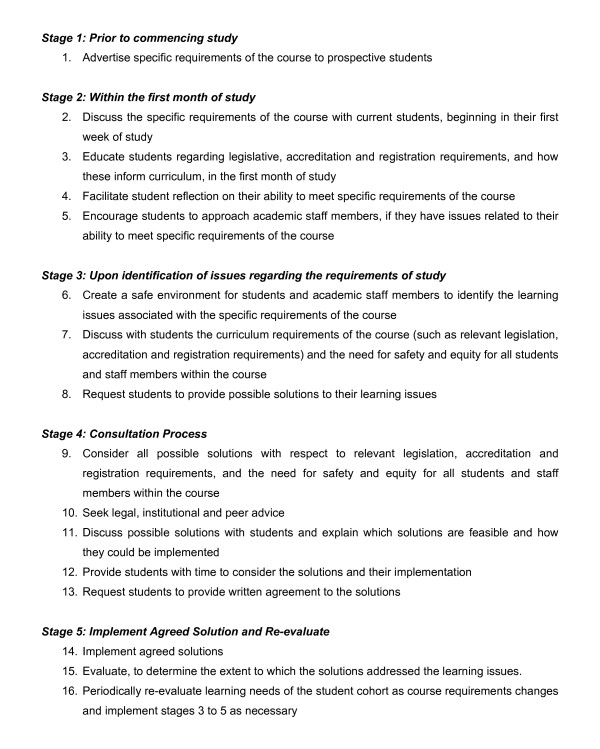
**The process used to resolve issues associated with a culturally-diverse cohort of students**.

Figure 3 provides a step-by-step process that was undertaken to resolve the cultural issues presented by the first intake of physiotherapy students at the University of Western Sydney. Stage 1 involves actively advertising the specific requirements of a course in publically-available literature pertaining to the course on offer. The requirements of disrobing in mixed gender classes and similarly being practiced on were overt messages delivered in University of Western Sydney publications, such as course handbooks, the website and University information days. Since our experiences with the 2010 intake of students, these messages have been refined to make them more explicit.

Stage 2 of the process involves educating new students regarding the nature of the physiotherapy profession and need for the specific requirements of study; teaching students how to be aware of their beliefs and how to identify when there is a mis-match between course requirements and their culture and beliefs. A safe environment must be created to ensure that students can articulate their concerns to educators if a mis-match is suspected. We used detailed and timely explanation of student expectations in the first weeks of university study, well before disrobing in practical classes commenced. This allowed students to self-identify issues related to expectations and discuss these in dedicated, private, educator consultation time. The small class size and the proactive nature of educators may well have contributed to creating a safe environment.

Stages 3 to 5 of the process involve formally resolving issues as they arise. This requires consideration of the legislative, accreditation and registration requirements of the profession, anti-discrimination legislation and the ethical principles of decision-making. Discussions with students and educators need to be undertaken in a culturally-sensitive manner; anti-discrimination and legal advice must be sought; and the process must be thoroughly documented. Educators' opinions need to be sought to determine the impact of possible solutions on their teaching methods and the probable impact on the individual and the student cohort as a whole. Students must be provided with adequate information and time to enable informed decisions and these decisions must be communicated in writing. Agreed solutions must be implemented and evaluated periodically, as the requirements of the course may change over time. Student's beliefs may also change over time, which may variably impact their ability to participate in learning activities.

### Agreed solutions

Discussion was undertaken with physiotherapy educators and students, which focused on the legislative, accreditation and registration requirements, anti-discrimination legislation and the ethical principles of decision-making. Students commented that they were unaware of the requirements of physiotherapy education, despite these being included in the first year physiotherapy curriculum. This curriculum has since been revised, with the legislative requirements and student expectations being made more explicit. Following these discussions, students understood the need to practice physiotherapy techniques on the opposite gender and to experience the techniques themselves, so that learning outcomes could be achieved. Students wore disposable gloves when practicing techniques on students of the opposite gender in university classes, thus eliminating skin contact with the opposite gender. Students also practiced these techniques on male relatives, as skin contact with relatives is permissible. Students disrobed in a semi-private area of the classroom, which was separated from other students by screens approximately 140 cm in height. These screens provided privacy while allowing educators to view student performance without exiting from the classroom. This arrangement contributed to safety of the whole student cohort, which was a major concern voiced by the educators. Any student was permitted to disrobe behind these screens. Educators agreed to not request students with issues related to cultural individualism to be demonstration models in practical classes. These models are required to disrobe and act as "patients" while the educator demonstrates physiotherapy techniques to the class. Students were also made aware that is highly likely that they will be taught by a male educator during their physiotherapy study. Students agreed that they would contribute to a physiotherapy policy regarding student supervision, which included information regarding cultural sensitivity.

### Evaluation

These solutions were implemented and evaluated following a semester of study. The students involved commented positively on the time and effort that was spent in accommodating their cultural beliefs. They were not aware of the legislative and accreditation requirements for physiotherapy, which underpins the physiotherapy curriculum. This comment added further justification to our curriculum approach, which uses legislative and accreditation requirements to explain curriculum design early in the first semester of physiotherapy study.

At the end of the semester, when specifically asked, both students responded favourably regarding their experience in practical classes. They thought that the implemented solutions worked well, although the location of the screened area could be improved, as it was situated in proximity to hand basins. The students felt uncomfortable whilst disrobed behind the screened area, when other students were washing their hands. This occurred on two occasions during the semester. We are currently planning new clinical skills teaching spaces, and the location and type of screens and the location of hand washing facilities have featured heavily in this planning process. We are in the process of purchasing portable, interlocking screens which can accommodate larger number of students if required and can be positioned in various locations depending on the layout of the teaching space. Moreover, the hand washing facilities will be located away from the plinths and screened areas, thus eliminating the issues identified by the students.

The educators involved in this process valued this experience as it challenged the way in which they taught physiotherapy skills. They commented that it was a useful exercise to revisit legislative and accreditation requirements to identify the key student competencies, on which the curriculum and teaching should be based. The physiotherapy program is currently building its full complement of physiotherapy staff, and it is likely that educators will not have taught such a culturally diverse student cohort. An education package for new educators and for physiotherapists who will be supervising students on clinical education placements is currently being developed to up skill educators on cultural issues associated with university and community-based teaching of students. University-based educators are also being proactive in identifying future issues that may be of relevance to these students, such as uniform requirements.

## Summary

The information presented in this article has focused on potential issues related to the delivery of physiotherapy curriculum in a culturally-diverse student cohort. These types of issues could potentially be present, and indeed relevant, in any health professional training program. The requirements related to the delivery of a physiotherapy program are generic concepts in that they are equally relevant to other health professions. The process that was undertaken to resolve the issues is also generalizable to other health professions.

## Competing interests

The authors declare that they have no competing interests.

## Authors' contributions

AB and CW were involved with identifying and resolving the issue. AB, CW and KGS developed the model and prepared the manuscript. All authors read and approved the final manuscript.

## Pre-publication history

The pre-publication history for this paper can be accessed here:

http://www.biomedcentral.com/1472-6920/11/34/prepub
